# Three new species of *Pseudophanias* Raffray from Japan and Taiwan Island, and synonymy of *Chandleriella* Hlaváč with *Pseudophanias* (Coleoptera, Staphylinidae, Pselaphinae)

**DOI:** 10.3897/zookeys.987.53648

**Published:** 2020-11-06

**Authors:** Shota Inoue, Shûhei Nomura, Zi-Wei Yin

**Affiliations:** 1 Entomological Laboratory, Graduate School of Bioresource and Bioenvironmental Sciences, Kyushu University, Fukuoka 819-0395, Japan; 2 The Kyushu University Museum, Fukuoka 812-8581, Japan; 3 Department of Zoology, National Museum of Nature and Science, 4-1-1, Amakubo, Tsukuba-shi, Ibaraki 305-0005, Japan; 4 Laboratory of Systematic Entomology, College of Life Sciences, Shanghai Normal University, 100 Guilin Road, Shanghai 200234, China

**Keywords:** claw morphology, East Asia, identification key, nomenclature, rove beetle, taxonomy, Tmesiphorini

## Abstract

The genus *Pseudophanias* Raffray, 1890 is discovered in Japan and Taiwan Island for the first time, with three new species: *P. yaimensis* Inoue, Nomura & Yin, **sp. nov.**, *P. nakanoi* Inoue, Nomura & Yin, **sp. nov.**, and *P. excavatus* Inoue, Nomura & Yin, **sp. nov.** It is the fifth tmesiphorine genus known from Japan and the first from Taiwan. The genus *Chandleriella* Hlaváč, 2000 is placed as a junior synonym of *Pseudophanias*, resulting in the following new combinations: *P. termitophilus* (Bryant, 1915), **comb. nov.**, and *P. yunnanicus* (Yin, 2019), **comb. nov.** A list of world species, and a key to East and South Asian representatives of *Pseudophanias* is provided.

## Introduction

The tribe Tmesiphorini Jeannel currently contains 30 extant genera worldwide (Yin et al. 2013a), among them the primarily Oriental genus *Pseudophanias* Raffray, 1890 was established for *P. malaianus* Raffray from Malaysia (Raffray 1890a, b). *Pseudophanias* was initially placed in the tribe Tyrini (Raffray 1905, 1908), then in Phalepsini (Jeannel 1949; Newton and Chandler 1989), and was most recently moved to Tmesiphorini (Chandler 2001). Currently, ten epigean species are known from the tropical areas in Southeast Asia (Raffray 1890a, b, 1895, 1905), and one cavernicolous species was described from central Nepal (Yin et al. 2015).

Hlaváč (2000) erected a new genus *Chandleriella* Hlaváč for *Lasinus termitophilus* Bryant from Borneo. He correctly recognized *Chandleriella* as a member of Tmesiphorini based on the presence of semi-circular sulci that enclose the antennal bases, but did not compare it to any members of the tribe. A second species of the genus, *C. yunnanica* Yin, was later described from southwestern China (Yin 2019), with two individuals of the type series associated with *Ectomomyrmex* ants.

Specimens of the genus *Chandleriella* have well-developed posterior tarsal claw and an overall elongate body form, whereas those of *Pseudophanias* have a strongly reduced posterior tarsal claw and a stouter body. However, after examining the type species of *Pseudophanias* (by the second author), and a vast collection of undescribed *Pseudophanias-Chandleriella*-like species, transitional states in both claw morphology and habitus were found. Nomura and Idris (2005) already discussed this problem in detail, but no further nomenclatural act was made. Consequently, the two genera cannot be distinguished reliably using current methods. On the other hand, Nomura and Idris (2005) also noted the similarities between *Pseudophanias* and the tribe Hybocephalini, but this relationship needs further investigation.

So far, four tmesiphorine genera have been recognized in Japan: *Saltisedes* Kubota, 1944, *Tmesiphorus* LeConte, 1849, *Tmesiphoromimus* Löbl, 1964, and *Raphitreus* Sharp, 1883 (Shibata et al. 2013; Yin et al. 2013b; Inoue et al. 2020), while none have been found from Taiwan. In this paper, we formally place *Chandleriella*, syn. n., as a junior synonym of *Pseudophanias* and describe three new species from Japan and Taiwan.

## Material and methods

The type specimens of *Pseudophanias* species in the Raffray’s Collection were examined by the second author at the Muséum National d’Histoire Naturelle, Paris, France (**MNHN**). The holotypes of *Pseudophanias yaimensis*, *P. nakanoi* and *P. excavatus* are deposited in the National Museum of Nature and Science, Tokyo, Japan (**NSMT**) and paratypes are deposited in NSMT, and the Kyushu University Museum, Fukuoka, Japan (**KUM**). Two paratypes of *Pseudophanias yaimensis* are temporally deposited in the Insect Collection of Shanghai Normal University, Shanghai, China (**SNUC**), and will be eventually housed in the National Museum of Natural Science, Taichung, Taiwan (**NMNS**).

The label data of the holotypes are quoted verbatim. A slash (/) was used to separate lines on the same label, and a double slash (//) was used to separate different labels on the same pin.

The specimens were soaked in distilled water overnight, and male genitalia were obtained by removing tergites and sternites VIII–IX. The male genitalia were soaked in cold 10% KOH for about 6 hours, and afterward they were washed in distilled water for 10 minutes. They were subsequently transferred to 50% ethanol for 2–5 minutes and then to 80% ethanol for 2 minutes. Finally, the male genitalia were soaked in 99% ethanol for 10 minutes and were then mounted in Euparal on a 5 × 10 mm micro-cover glass. The micro-cover glass with male genitalia was glued onto a paper card (5 × 7 mm) and pinned under the specimen (Maruyama 2004). The specimens were also examined using a scanning electron microscope (SEM; Hitachi SU-3500) at the Center for Advanced Instrumental and Educational Supports, Faculty of Agriculture, Kyushu University. For the SEM observations, all examined materials were coated with gold by Ion Sputter (Ion sputter; Hitachi MC1000) and examined at 5.00 kV and 30 Pa.

The following abbreviations were applied:

**AL** length of the dorsally visible part of the abdomen along the midline;

**AW** maximum width of the abdomen;

**EL** length of the elytra along the suture;

**EW** maximum width of the elytra;

**HL** length of the head from the anterior clypeal margin to the occipital constriction;

**HW** width of the head across the eyes;

**PL** length of the pronotum along the midline;

**PW** maximum width of the pronotum.

Length of the body (BL) was a combination of HL + PL + EL + AL. All measurements are recorded in millimeters (mm).

## Taxonomy

### Genus Pseudophanias

Taxon classificationAnimaliaColeopteraStaphylinidae

Raffray, 1890

DEA6E159-D8DE-521D-AB28-A51F627A798F

Pseudophanias Raffray, 1890a: 161. Type species: Pseudophanias malaianus Raffray, 1890b: 214 (by subsequent monotypy).
Chandleriella Hlaváč, 2000: 91, syn. nov. Type species: Lasinus termitophilus Bryant, 1915: 300 (by original monotypy).

#### Revised diagnosis.

Members of the genus *Pseudophanias* can be distinguished from all other genera of the Tmesiphorini by a combination of the following characteristics: body form strongly stout to markedly elongate; male antennae usually with modified antennomeres 3–10, or 11 alone; greatly reduced maxillary palpi with fusiform palpomere 4; distinct paratergites on abdomen; tergite IV longest to subequal in size to tergite V; aedeagus usually tuberculate in shape, rarely bulbous.

#### Distribution.

Indonesia, Malaysia, Singapore, China, Japan, Nepal.

### Key to *Pseudophanias* of East and South Asia

**Table:** 

1	Head coarsely punctate (Fig. 11A). Antennae short (ratio of body length to antennal length = 1:<0.5), antennomeres each distinctly transverse, antennomeres 5 to 11 modified to form clasping in male (Fig. 10B, C) (China: Taiwan)	***P. excavatus* Inoue, Nomura & Yin, sp. nov.**
–	Head finely punctate. Antennae elongate (ratio of body length to antennal length = 1:>0.5), antennomeres each elongate to subquadrate, antennomeres 7, 9, or 11 modified to form various shape in male	**2**
2	Pronotum with median longitudinal carina (Indonesia, Sumatra)	***P. termitophilus* (Bryant)**
–	Pronotum without median longitudinal carina	**3**
3	Antennomeres each distinctly elongate, male antennomere 7 expanded. Male profemora excavated at bases (Nepal: Pokhara)	***P. spinitarsis* Yin, Coulon & Bekchiev**
–	Antennomeres each elongate to subquadrate, male antennomere 7 unmodified. Profemora evenly narrowing at bases in both sex	**4**
4	Antennomere 9 obliquely expanded laterally, antennomere 11 angularly expanded at lateral margins in male (Fig. 6B). Abdominal tergite IV with long discal carinae (Fig. 7F) (Japan: Ryûkyû, Kyûshû)	***P. nakanoi* Inoue, Nomura & Yin, sp. nov.**
–	Antennomere 9 simple, antennomere 11 modified. Abdominal tergite IV with short discal carinae	**5**
5	Body length 3.50–3.90 mm (female: 3.83–3.85 mm). Antennomere 11 enlarged to form bowl-like structure in male. Pronotum without conical spine (China: Yunnan)	***P* . *yunnanicus* (Yin)**
–	Body length 2.16–2.32 mm (female: 2.20–2.32 mm). Antennomere 11 angulate at anterolateral margins in male (Fig. 2B). Pronotum with conical spine just anterior of median fovea (Fig. 2A) (Japan: Ryûkyû; China: Taiwan)	***P. yaimensis* Inoue, Nomura & Yin, sp. nov.**

### Pseudophanias yaimensis

Taxon classificationAnimaliaColeopteraStaphylinidae

Inoue, Nomura & Yin sp. nov.

897F5BB0-F37F-5E33-93A8-13DA9D806DA6

http://zoobank.org/81ADE876-E798-467A-8625-03721012EF1B

**Figure 1.**
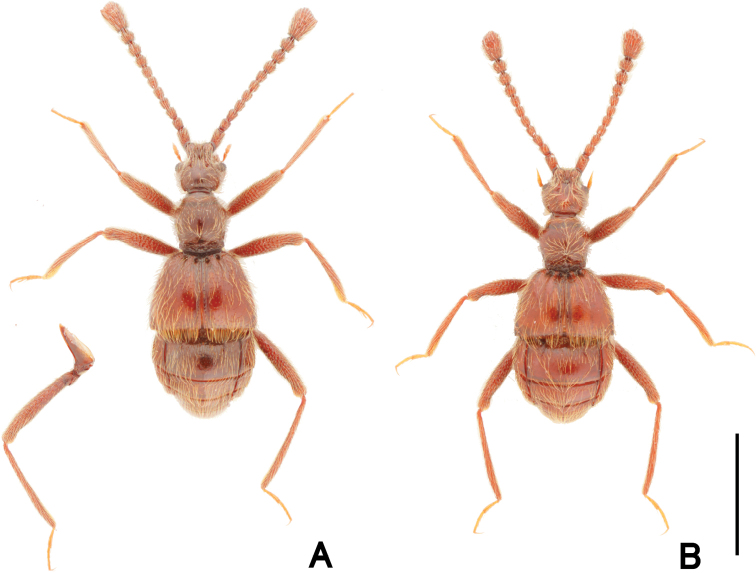
Dorsal habitus of *Pseudophanias yaimensis***A** male **B** female. Scale bar: 1.0 mm.

**Figure 2.**
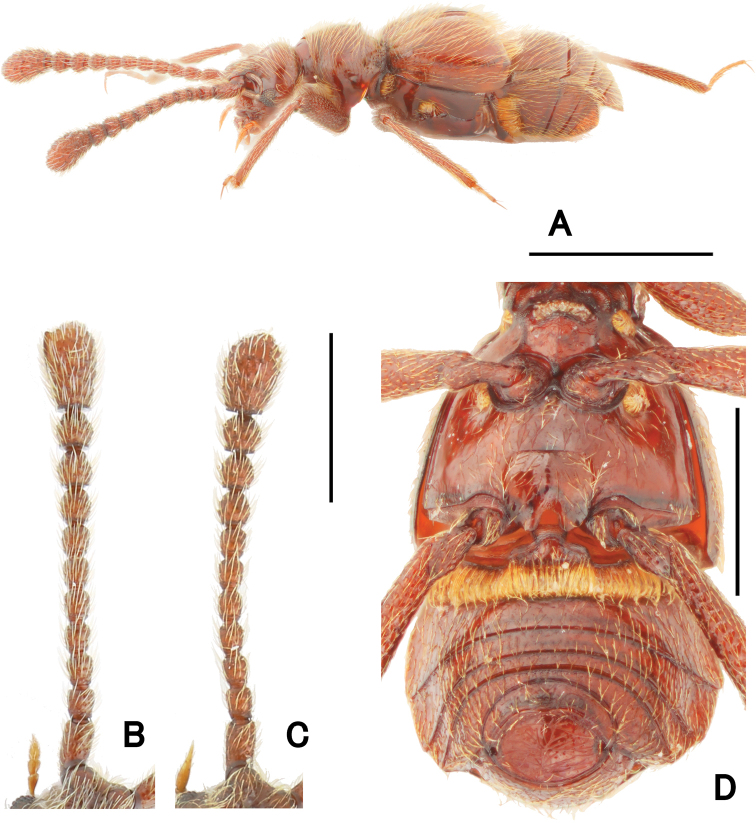
Morphological details of *Pseudophanias yaimensis***A** habitus in lateral view **B** male antenna **C** female antenna **D** male metaventrite. Scale bars: 1.0 mm (**A**); 0.5 mm (**D**); 0.2 mm (**B, C**).

**Figure 3.**
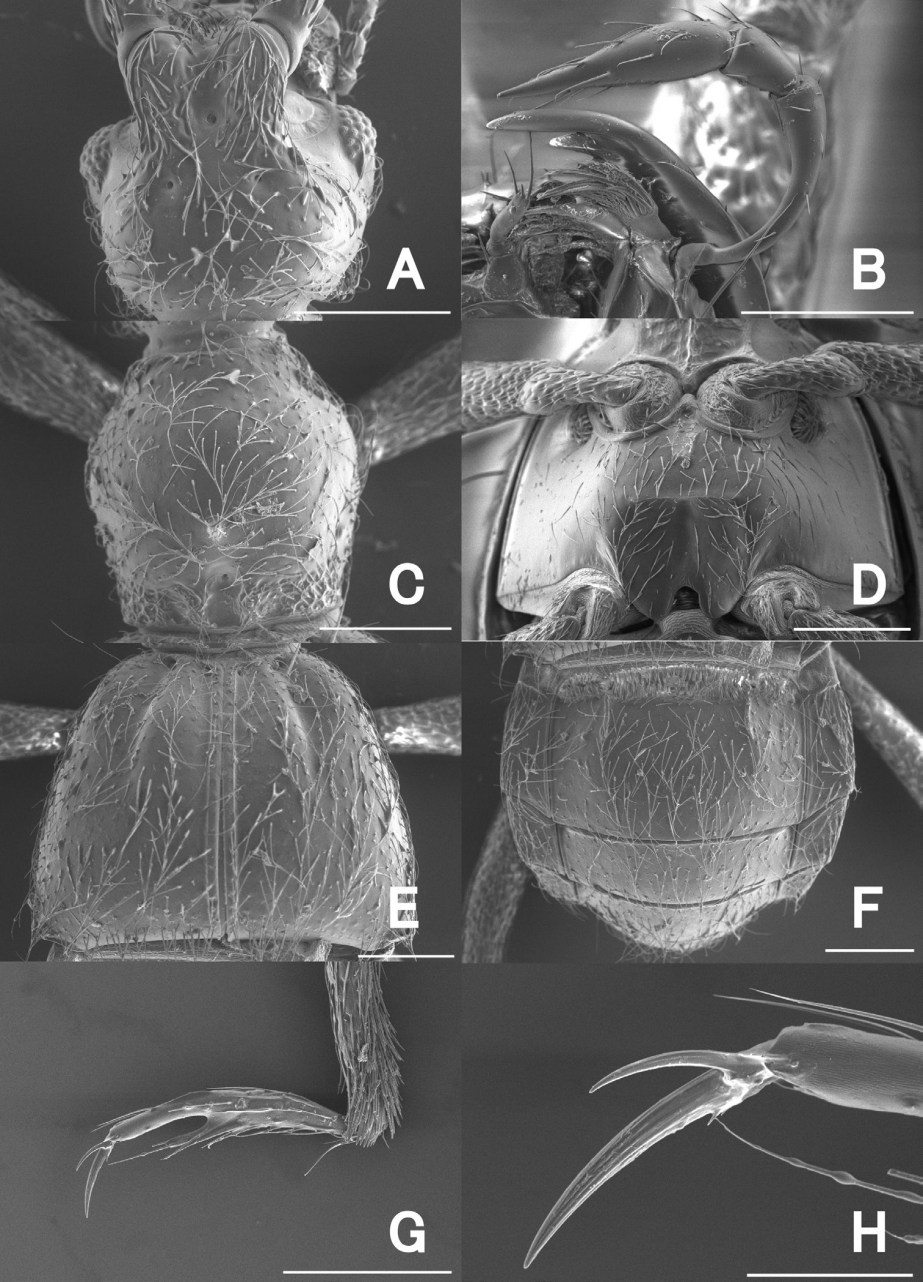
SEM images of *Pseudophanias yaimensis*, male **A** head **B** maxillary palp **C** pronotum **D** metaventrite **E** elytra **F** abdomen **G** mesotarsus **H** mesotarsal claws. Scale bars: 0.2 mm (**A, C–G**); 0.1 mm (**B**); 0.05 mm (**H**).

**Figure 4.**
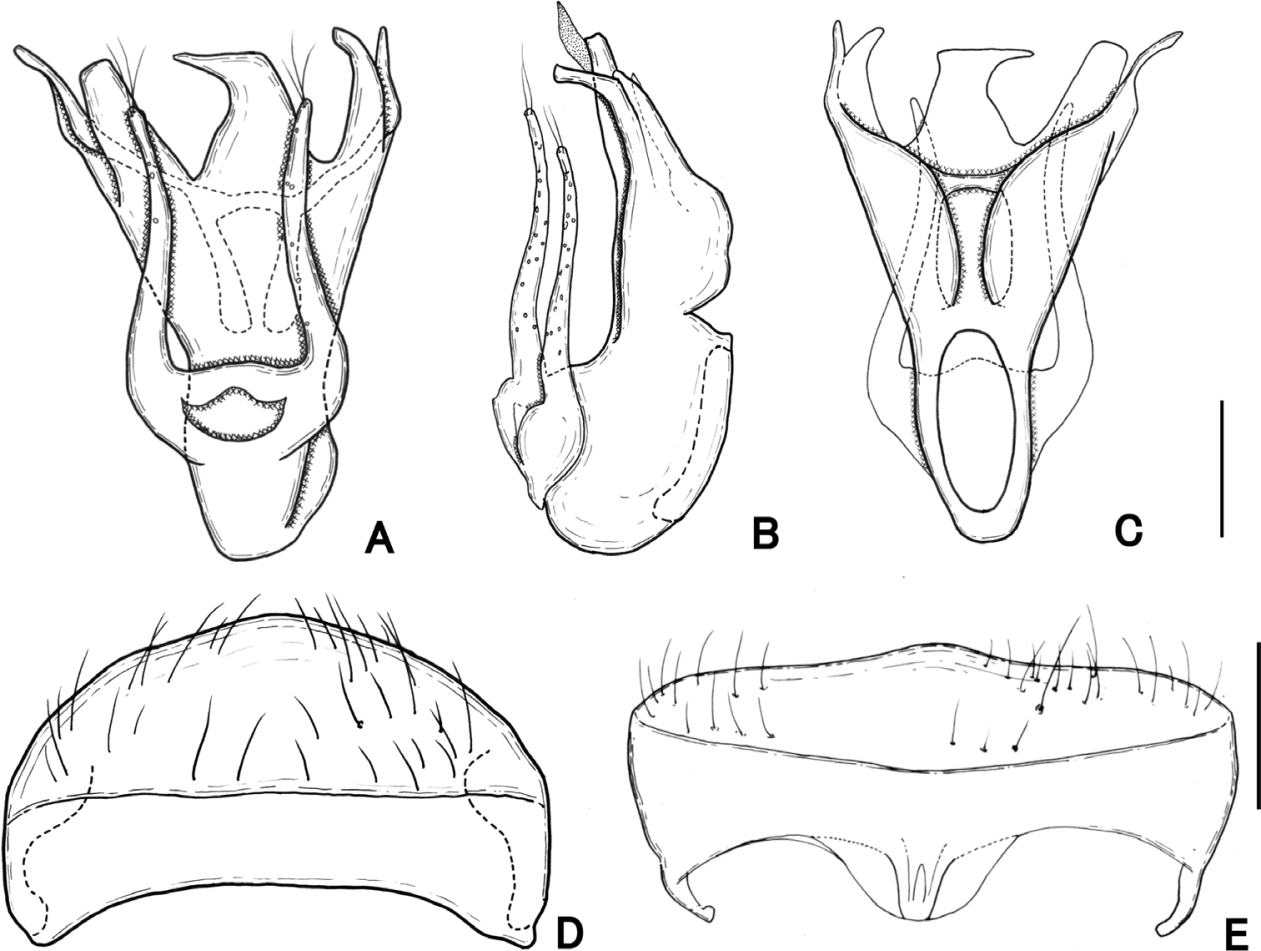
Male genitalia of *Pseudophanias yaimensis***A** ventral view **B** lateral view **C** dorsal view **D** tergite VIII **E** sternite VIII. Scale bars: 0.1 mm.

Figs 1 , 2 , 3 , 4 [Japanese name: Yaima-tsumugata-arizukamushi]

#### Type material.

***Holotype*** (NSMT): ♂, “Japan: [Ryûkyû], Ishigaki- / jima, Takeda-rindô 23 X 2007, Teruaki Ban leg. // HOLOTYPE (red) /♂, *Pseudophanias yaimensis* sp. nov., / det. Inoue, Nomura & Yin, 2020” ***Paratypes***: Japan: 1 ♀, [Ryûkyû], Okinawa ken, Ishigaki-jima Is., Mt. Omoto-dake, 16 VIII 1991, K. Ogata leg. (NSMT); 1 ♂, Ishigaki-jima Is., Mt. Omoto-dake, (FIT), 14–20 V 2002, S. Hori leg. (NSMT); 1 ♂, 1 ♀, China: Taiwan, Taichung Co. (台中县), Guguan (谷关), 1238 m, 24.180691N, 120.944213E, 29 III 2015, local collector, nest of *Nasutitermes parvonasutus* (SNUC). Each paratype pinned with the following label: “PARATYPE (yellow) / ♂ (or ♀), *Pseudophanias yaimensis* sp. nov., / det. Inoue, Nomura & Yin, 2020”.

#### Diagnosis.

*Pseudophanias yaimensis* is most similar to the Sumatran *P. robustus* Raffray, 1904, but can be distinguished by the distinctly smaller body size (3.00–3.20 mm in *P. robustus*), the angulate antennomere 11 at anterolateral margins in the male, the finely punctate head and pronotum, and the shorter discal carinae on tergite IV.

#### Description.

**Male** (Figs 1A, 2A). Body length 2.16–2.32 mm. Dorsal surface polished and weakly shining, with dense setae. ***Head*** (Fig. 3A) about as long as wide, HL 0.46 mm, HW 0.42–0.44 mm, same size as pronotum, nearly hexagonal, with dense setae; frontal rostrum with short longitudinal sulcus including large fovea; antennal tubercles prominent, with dense punctures which gradually disappear towards vertex; vertex polished, finely punctate, with pair of foveae; frontal, vertexal foveae glabrous; eyes prominent; postocular margins three times longer than length of eyes. Maxillary palpi (Fig. 3B) symmetrical; palpomere 1 minute; palpomere 2 elongate, narrowed in basal half; palpomere 3 small, widest at apices; palpomere 4 fusiform. ***Antennae*** (Fig. 2B) elongate, 1.42 mm in length; antennal club formed by apical antennomere alone; antennomere 1 thick, elongate, 1.5 times longer than 2; 2 slightly longer than wide; 3–11 successively widened towards apices; 3–7 each slightly elongate; 8–10 quadrate; 11 enlarged, roundly broadened towards apices in inner margin, straightened towards apices in outer margin; each apical half of outer margin distinctly carinate, with angulate spine at anterolateral part. ***Pronotum*** (Fig. 3C) slightly longer than wide, PL 0.48 mm, PW 0.46 mm; widest at anterior one-third, weakly constricted from widest point towards base, polished, with coarse punctures along posterior margin, with a median and pair of lateral antebasal foveae, with distinct conical spine just anterior of median fovea. ***Metaventrite*** (Figs 2D, 3D) finely punctate, moderately convex, but area just above metaventral apex impressed; that impression nearly trapezoidal, half as long as metaventral length, occupying 1/4 metaventral width; anterior margin of that impression straight, distinct. ***Elytra*** (Fig. 3E) nearly trapezoidal, widest near posterior 1/4 much wider than long, EL 0.60–0.62 mm, EW 0.85–0.94 mm; dorsal surface polished, with thin, long setae, finely punctate; each elytron with two basal foveae; discal stria shallow, extending from basal fovea placed middle to posterior half. ***Legs*.** All legs elongate and slender; femora each broadest near middle; tibiae each slightly broadened to apex, with dense yellow setae at apex; protibiae and mesotibiae nearly straight; metatibiae longest; tarsi each elongate, with tarsomeres 2 about as long as tarsomeres 3; mesotarsi (Fig. 3G) modified, broadest; tarsomeres 3 each with projection of 2/3 length of entire tarsomere; tarsal claws (Fig. 3H) each asymmetrical; posterior claws thin, short. ***Abdomen*** (Fig. 3F) wider than long, widest at tergite IV, AL 0.62–0.76 mm, AW 0.85–0.90 mm; tergite IV longest, twice as long as tergite V, with pair of short longitudinal carinae about one-fifth as long as tergal length, with setose depression at base. Tergite and sternite VIII as in Fig. 4D, E. ***Aedeagus*** (Fig. 4A–C) 0.39–0.40 mm in length, well-sclerotized, asymmetrical; parameres elongate, reaching near apex of median lobe; each paramere with three setae at apex; median lobe strongly widened towards apex in dorso-ventral view, constricted at median part in lateral view; ventral side of apical part asymmetrical, weakly sclerotized, nearly formed three pronged fork; medioapical part bended laterally; dorsal side of apical part asymmetrical, bifurcate from median part; endophallus indistinct.

**Female** (Fig. 1B). BL 2.20–2.32 mm; HL 0.40–0.52 mm; HW 0.42–0.45 mm; PL 0.46–0.49 mm; PW 0.44–0.46 mm; EL 0.58–0.63 mm; EW 0.78–0.89 mm; AL 0.70–0.76 mm; AW 0.86–0.90 mm. Antennae (Fig. 2C) with antennomeres 11 unmodified, each ovoid, without carina and angulate spine; eyes smaller than male; mesotarsi without projection.

#### Etymology.

Ishigaki Island, where the type locality of this species was discovered, is a part of the Yaeyama Islands. This specific epithet refers to Yaima which is a local dialect of the Yaeyama Islands.

#### Distribution.

Japan (Ryûkyû: Ishigaki-jima Is.), China (Taiwan).

#### Biology.

Two paratypes from Taiwan were collected with the termite *Nasutitermes parvonasutus* Nawa, 1911. In Japan, one paratype was collected using a Flight Interception Trap (FIT).

#### Remarks.

This species is distributed in Yaeyama-shotô Islands, Japan and Taiwan, China. The two localities are close to each other and shared the same fauna for some insect groups. The two populations show slight difference in the morphology of the aedeagus. The lateral projections of the median lobe of the population of Taiwan are relatively longer and narrower than those of the population of Yaeyama. But the general appearance and especially the male sexual characters are otherwise almost identical. Therefore, we treat such a difference as interspecific variation.

The Taiwanese specimens were collected from a nest of the *Nasutitermes parvonasutus* termite. However, the Japanese specimens were collected from leaf litter samples or by FIT. Some pselaphine species are known to live under the bark and rotten wood with termites. Thus, more information is needed to recognize the possible termitophyly of the new species.

### Pseudophanias nakanoi

Taxon classificationAnimaliaColeopteraStaphylinidae

Inoue, Nomura & Yin sp. nov.

FB066C47-209D-5E66-B62D-E2F86E8F6361

http://zoobank.org/6782BACF-BBD3-423C-8A48-5EAD6B3645C1

**Figure 5.**
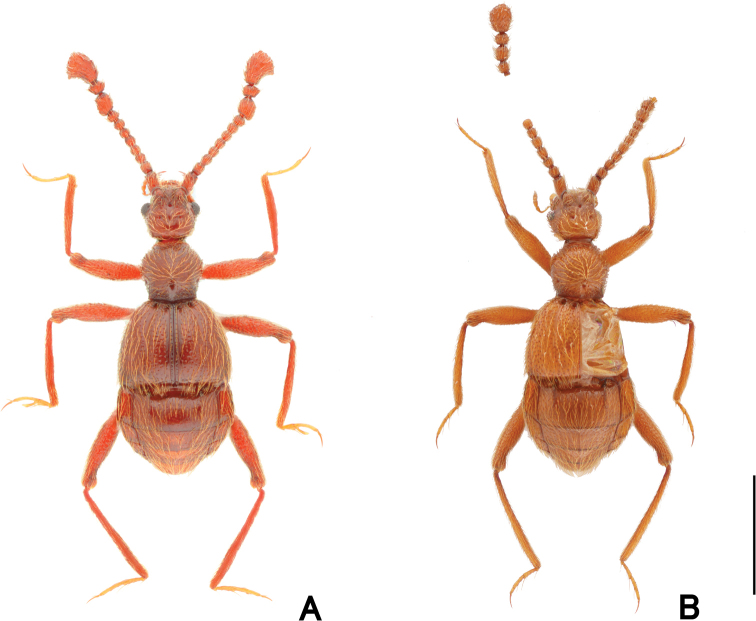
Dorsal habitus of *Pseudophanias nakanoi***A** male **B** female. Scale bar: 1.0 mm.

**Figure 6.**
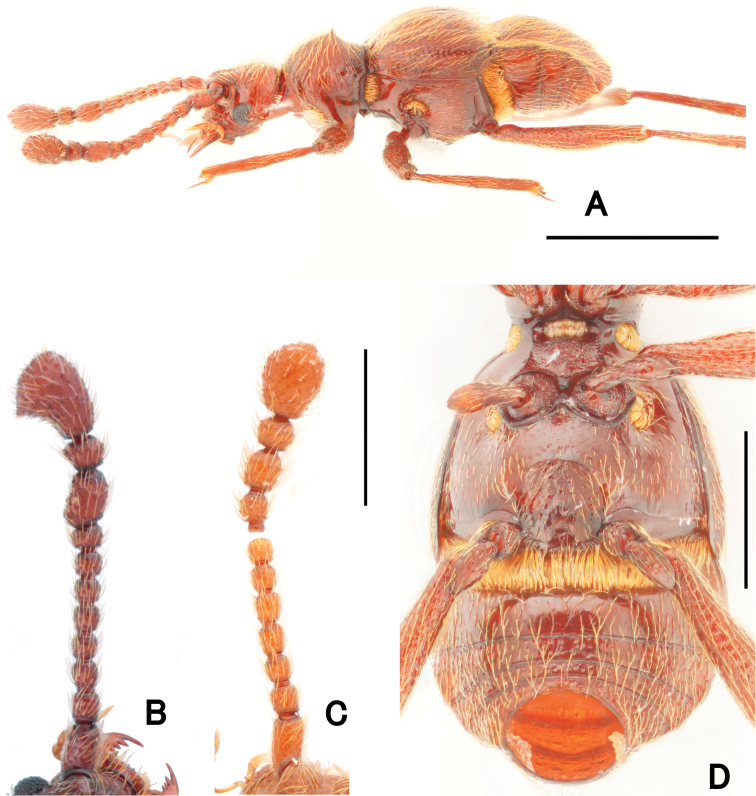
Morphological details of *Pseudophanias nakanoi***A** habitus in lateral view **B** male antenna **C** female antenna **D** male metaventrite. Scale bars: 1.0 mm (**A**); 0.5 mm (**D**); 0.2 mm (**B, C**).

**Figure 7.**
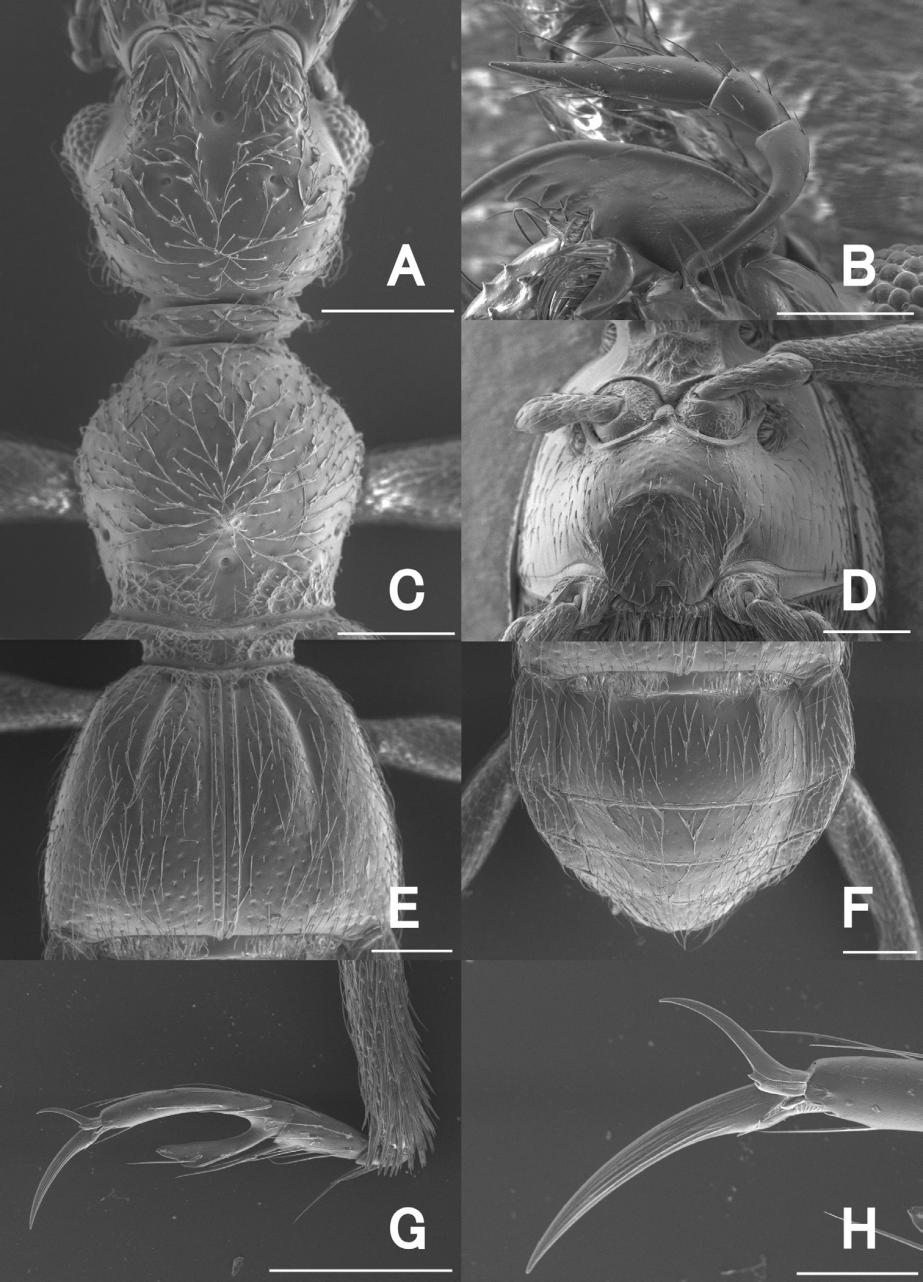
SEM images of *Pseudophanias nakanoi*, male **A** head **B** maxillary palp **C** pronotum **D** metaventrite **E** elytra **F** abdomen **G** mesotarsus **H** mesotarsal claws. Scale bar: 0.2 mm (**A, C–G**); 0.1 mm (**B**); 0.05 mm (**H**).

**Figure 8.**
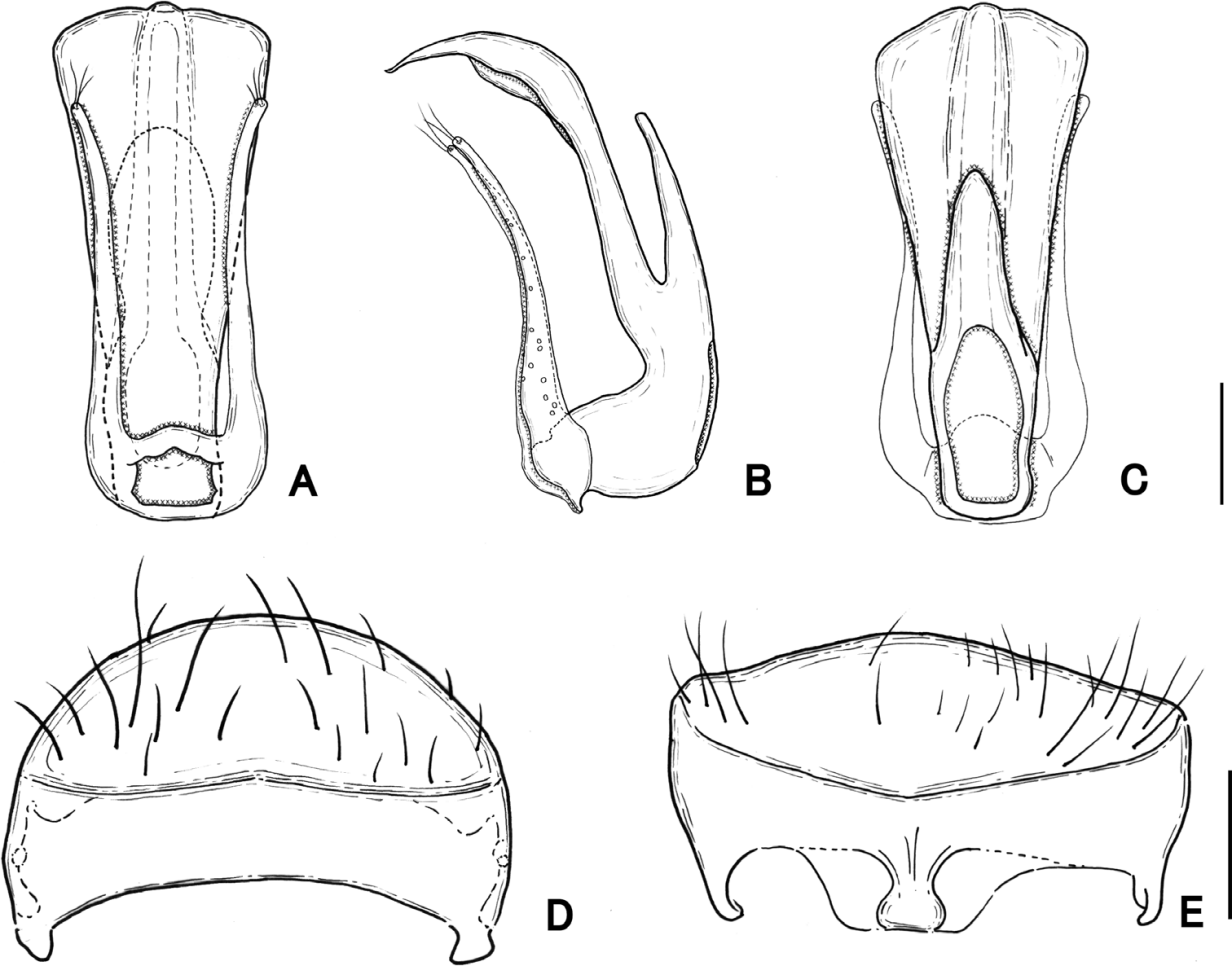
Male genitalia of *Pseudophanias nakanoi***A** ventral view **B** lateral view **C** dorsal view **D** tergite VIII **E** sternite VIII. Scale bars: 0.1 mm.

Figs 5 , 6 , 7 , 8 [Japanese name: Nakano-tsumugata-arizukamushi]

#### Type material.

***Holotype*** (NSMT): ♂, “屋久島 小楊枝林道, alt. 190 m. / [Yakushima Is.], Japan: / Kagoshima-ken, Kumage-gun, / Yakushima-chô, Koyôji-rindô / 30°17'36"N, 130°25'30"E, / 21 IX 2018, F. Nakano leg. // HOLOTYPE (red) /♂, *Pseudophanias nakanoi* sp. nov., / det. Inoue, Nomura & Yin, 2020” ***Paratype***: Japan: 1 ♀, [Ryûkyû; Tokara-rettô Isls.], Kagoshima-ken, Toshima-mura, Nakano-shima Is, Sato, 7–9 VII 2019, (leaf litter), N. Tsuji leg., with collecting permission of Toshima-mura Village (NSMT). Paratype pinned with the following label: “PARATYPE (yellow) / ♀, *Pseudophanias nakanoi* sp. nov., / det. Inoue, Nomura & Yin, 2020”.

#### Diagnosis.

*Pseudophanias nakanoi* is similar to *P. clavatus* Raffray, 1904, but *P. nakanoi* can be distinguished from the latter by its modified antennal clubs, which are formed by three apical antennomeres, and the finely punctate head and pronotum.

#### Description.

**Male** (Figs 5A, 6A). Body length 2.59 mm. Dorsal surface densely covered with long setae. ***Head*** (Fig. 7A) as long as wide, HL 0.52 mm, HW 0.52 mm, nearly trapezoidal; frontal rostrum broad, short, strongly sulcate along midline, with large fovea in frontal sulcus; antennal tubercles distinct, with dense punctures which gradually disappear towards vertex; vertex polished, finely punctate, with pair of foveae; frontal, vertexal foveae glabrous; eyes large, weakly prominent; postocular margins two times longer than length of eyes. ***Antennae*** (Fig. 6B) moderately elongate, 1.36 mm in length; antennal clubs formed by apical three antennomeres; antennomeres 1 thick, elongate, 1.5 times longer than 2; 2 slightly longer than wide; 3–7 nearly moniliform; 8 transverse; 9–11 enlarged, slightly excavated in outer margin; 9 nearly ovoid, each with smooth area on ventral surface; 10 transverse, half as long as 9, each with smooth area on ventral surface; 11 enlarged, widest at apical 1/3, strongly produced outward, roundly broadened to apices in inner margin, each with smooth area on ventral surface. Maxillary palpi (Fig. 7B) symmetrical; palpomeres 1 minute; palpomeres 2 elongate, narrowed in basal halves; palpomeres 3 small, widest at apices; palpomeres 4 fusiform. ***Pronotum*** (Fig. 7C) slightly wider than long, PL 0.53 mm, PW 0.55 mm; widest near anterior 1/3, then narrowed anteriorly, posteriorly, with coarse punctures along posterior margin, with a median and pair of lateral antebasal foveae, with distinct conical spine just anterior of above median fovea. ***Metaventrite*** (Figs 6D, 7D) finely punctate, moderately convex, but just above metaventral apex roundly impressed; that impression half as long as metaventral length, occupying 1/4 metaventral width; anterior margin of that impression moderately rounded, distinct. ***Elytra*** (Fig. 7E) nearly subtrapezoidal, widest near posterior 1/4 much wider than long, EL 0.72 mm, EW 0.99 mm, with dense setae, coarsely punctate, each elytron with two basal foveae; discal stria shallow, extending from median fovea to posterior 1/3. ***Legs*.** All legs moderately elongate and slender; femora each broadest near middle; tibiae each slightly broadened to apices, with dense yellow setae at apices; protibiae and mesotibiae nearly straight; metatibiae longest; tarsi each elongate, with tarsomeres 2 two-thirds as long as 3; mesotarsi (Fig. 7G) modified, broadest; tarsomeres 3 each with projection of 3/4 length of entire tarsomeres; tarsal claws (Fig. 7H) asymmetrical; anterior claws long; posterior claws thin, short. ***Abdomen*** (Fig. 7F) wider than long, widest at tergite IV, AL 0.82 mm, AW 1.03 mm; tergite IV longest, twice as long as tergite V, with pair of longitudinal carinae extending to posterior half, with mediobasal setose depression distinct. Tergite and sternite VIII as in Fig. 8D, E. ***Aedeagus*** (Fig. 8A–C) 0.46 mm in length, well-sclerotized, symmetrical in dorso-ventral view; parameres extremely elongate, reaching near apical fourth, each with three setae at apex; median lobe broad at base, split into ventral and dorsal lobes in lateral view; ventral lobe broadened from base toward apex in dorso-ventral view, curved ventrally and narrowed apically in lateral view; dorsal lobe terminated apical 1/3 of ventral lobe; endophallus indistinct.

**Female** (Fig. 5B). BL 2.56 mm; HL 0.49 mm; HW 0.52 mm; PL 0.53 mm; PW 0.57 mm; EL 0.73 mm; EW 1.00 mm; AL 0.81 mm; AW 1.03 mm. Antennae (Fig. 6C) unmodified, nearly moniliform, successively broadened apically; antennomeres 11 largest; eyes smaller than in male; mesotarsi without projection.

#### Etymology.

The new species is named after Mr Fumitaka Nakano, the original collector of the holotype.

#### Distribution.

Japan (Ryûkyû: Tokara-rettô Isls.; Kyûshû: Yakushima Is.)

#### Biology.

The holotype was collected from a dead tree of the family Fagaceae, and the paratype female was collected from leaf litter.

### Pseudophanias excavatus

Taxon classificationAnimaliaColeopteraStaphylinidae

Inoue, Nomura & Yin sp. nov.

190D674F-79B3-5837-8011-AF4CAE51FF2B

http://zoobank.org/E9C17AEF-CEA2-45DB-A952-52905F0943BE

**Figure 9.**
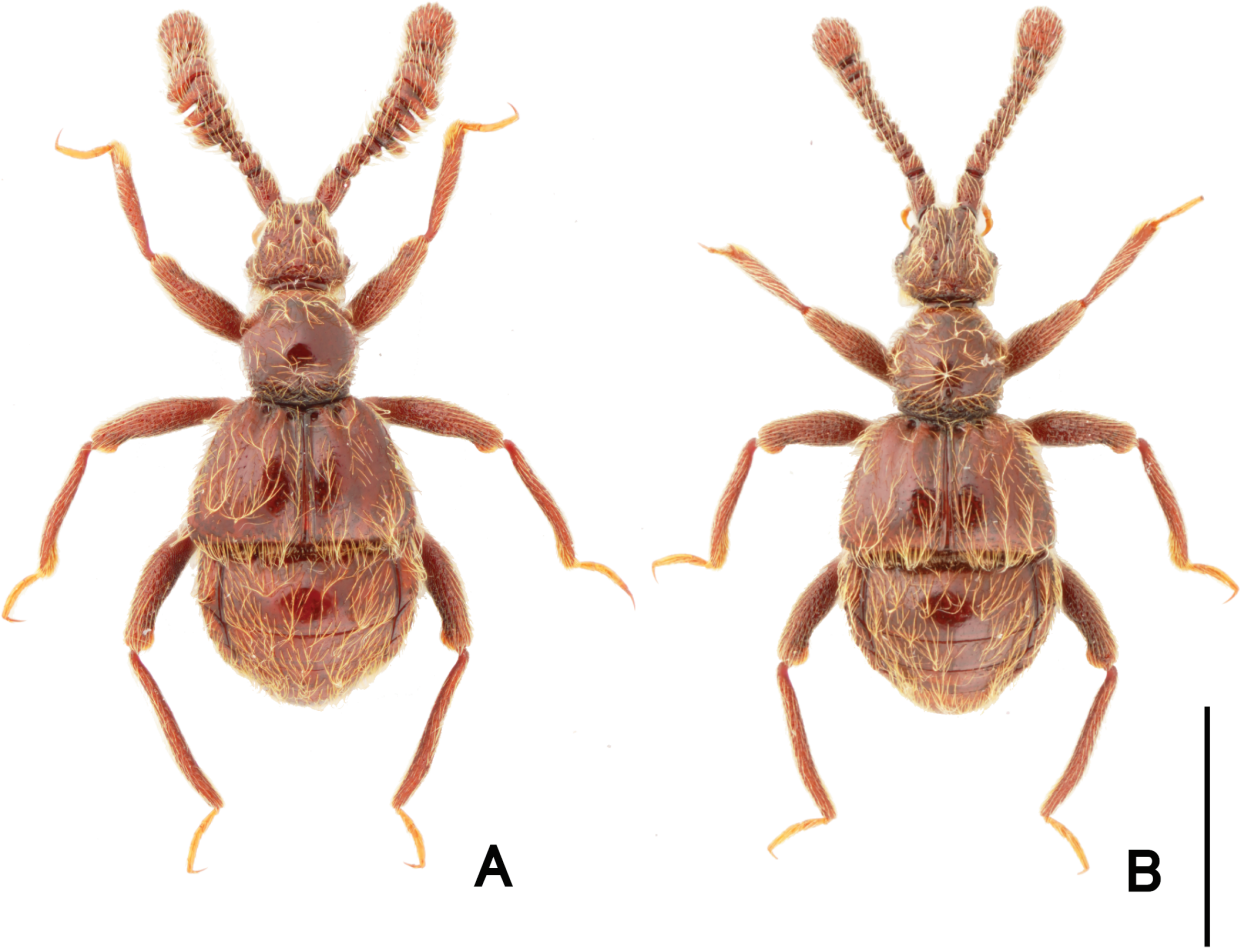
Dorsal habitus of *Pseudophanias excavatus***A** male **B** female. Scale bar: 1.0 mm.

**Figure 10.**
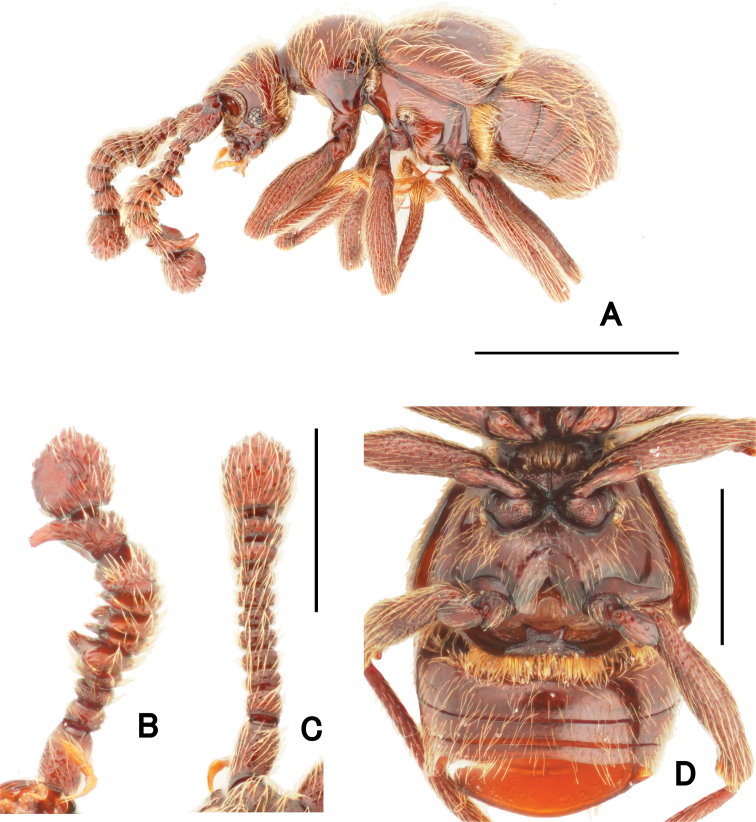
Morphological details of *Pseudophanias excavatus***A** habitus in lateral view **B** male antenna **C** female antenna **D** male metaventrite. Scale bars: 1.0 mm (**A**); 0.5 mm (**D**); 0.2 mm (**B, C**).

**Figure 11.**
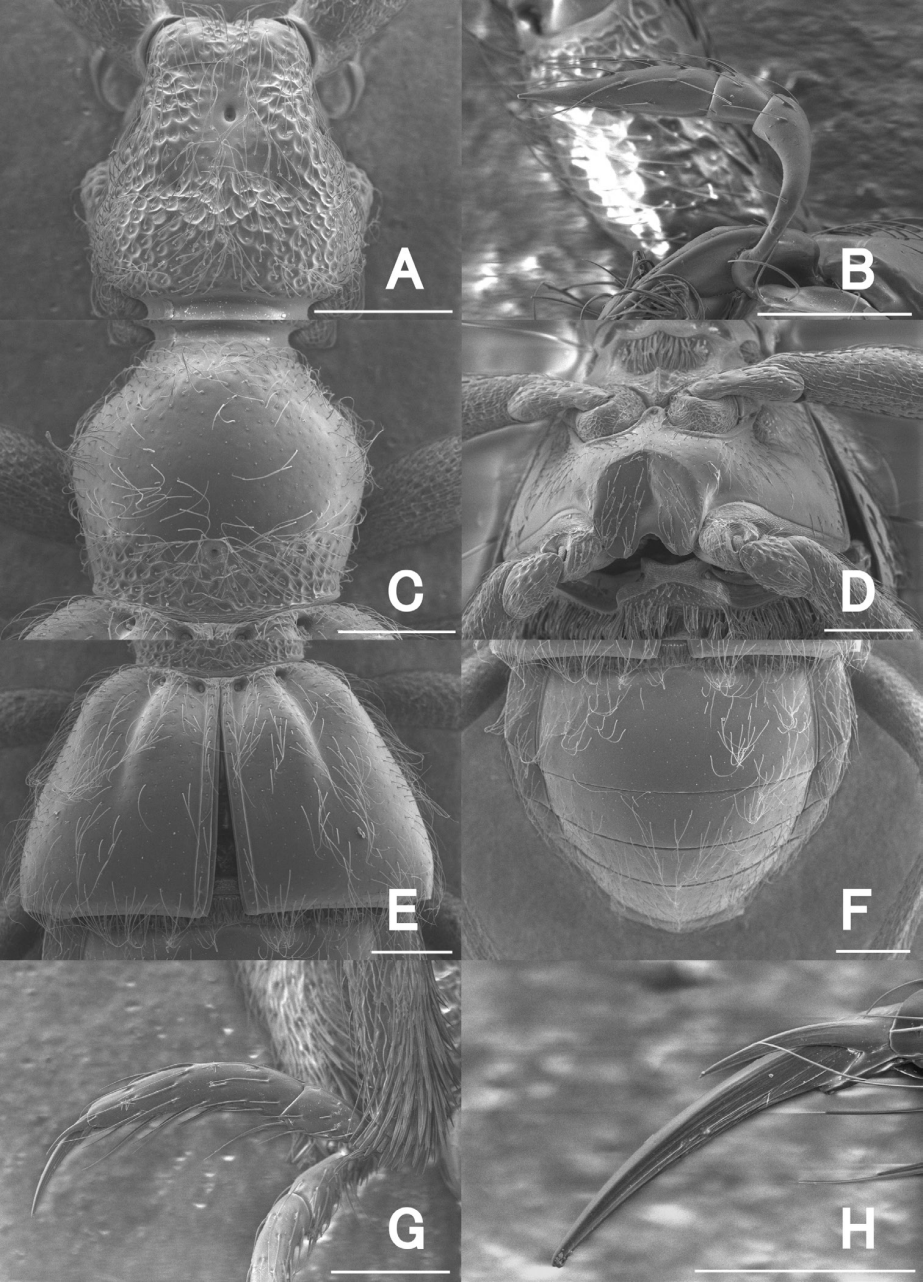
SEM images of *Pseudophanias excavatus*, male **A** head **B** maxillary palp **C** pronotum **D** metaventrite **E** elytra **F** abdomen **G** protarsus **H** protarsal claws. Scale bars: 0.2 mm (**A, C–F**); 0.1 mm (**B, G**); 0.05 mm (**H**).

**Figure 12.**
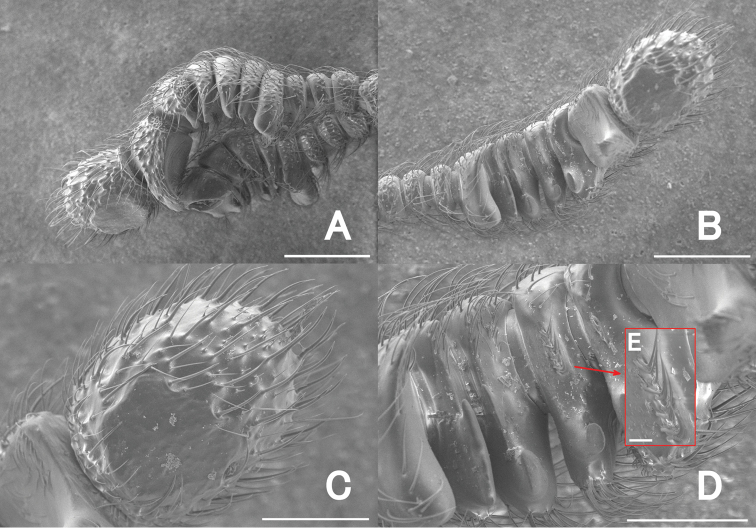
Male antenna of *Pseudophanias excavatus* by SEM images **A** lateral view **B** ventral view **C** antennomere 11 in ventral view **D** antennomeres 5–10 in ventral view **E** special setae on antennomere VIII in ventral view. Scale bars: 0.2 mm (**A, B**); 0.1 mm (**C, D**); 0.01 mm (**E**).

**Figure 13.**
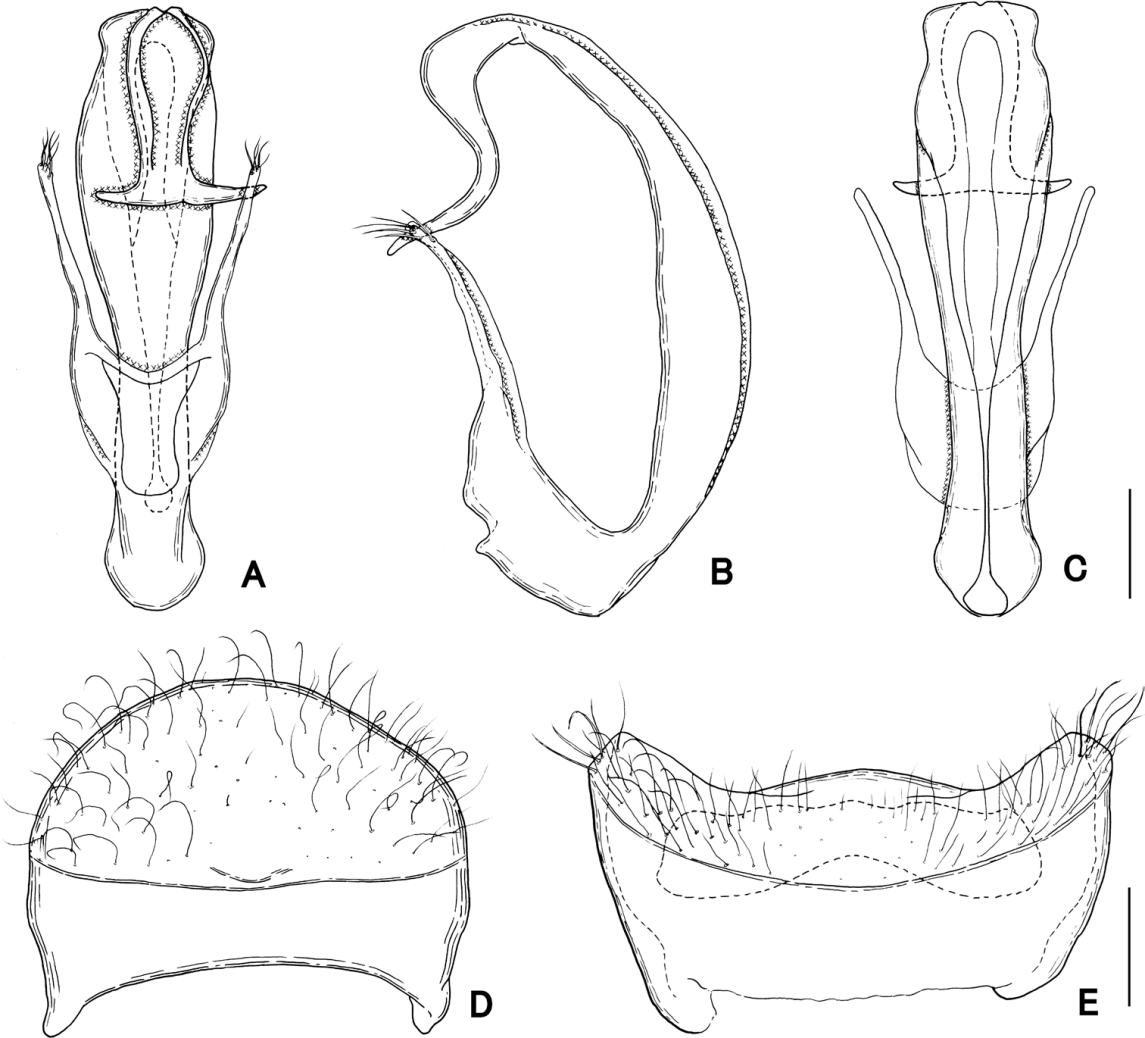
Male genitalia of *Pseudophanias excavatus***A** ventral view **B** lateral view **C** dorsal view **D** tergite VIII **E** sternite VIII. Scale bars: 0.1 mm.

Figs 9 , 10 , 11 , 12 , 13

#### Type material.

***Holotype*** (NSMT): ♂, “Tengshih (1400 m, litter) / Kaosiung Hsien / [M-Taiwan] / 台湾高雄縣藤枝 / 20–22. iv. 2001, H. Sugaya leg. // HOLOTYPE (red) /♂, *Pseudophanias excavatus* sp. nov., / det. Inoue, Nomura & Yin, 2020” ***Paratypes***: (NSMT, KUM, NMNS) 1 ♂, 3 ♀, same data as holotype; 6 ♂, 1 ♀, same data as holotype, but 29–30 IV 2001; 1 ♂, 2 ♀, same data as holotype, but 30 IV 2001. Each paratype pinned with the following label: “PARATYPE (yellow) / ♂ (or ♀), *Pseudophanias excavatus* sp. nov., / det. Inoue, Nomura & Yin, 2020”.

#### Diagnosis.

This species is readily distinguished from other members of *Pseudophanias* by the clasping formed antennae in the male, frontal sulcus indistinct, and the rounded pronotum.

#### Description.

**Male** (Figs 9A, 10A). Body length 2.21–2.48 mm. Dorsal surface with dense setae.

***Head*** (Fig. 11A) as long as wide, HL 0.45–0.50 mm, HW 0.43–0.50 mm, densely punctate, with dense, long setae; frontal rostrum broad, with frontal fovea nude; antennal tubercles distinct; vertex flat, with pair of glabrous foveae; eyes prominent, small; occiput with dense setae; postocular margin two times longer than eyes; small areas just posterior to U-shaped setose sulci finely punctate. ***Antennae*** (Figs 10B, 12A–E) strongly modified, 0.98–1.12 mm in length; antennomeres 1 elongate, as long as 2–4 combined; 2–4 each transverse, successively shorter; 5–11 strongly excavated on ventral side, with tufts of setae on ventral surface, modified to form clasping, each excavated on ventral surface; 5–9 each two times wider than 4, each transverse; 9 longer than 8; 10 as long as 9, asymmetrical; outer side strongly produced ventrally in 9; 11 enlarged, with glabrous areas on ventral side. Maxillary palpi (Fig. 11B) symmetrical; palpomeres 1 minute; palpomeres 2 elongate, narrowed in basal halves; palpomeres 3 small, widest at apices; palpomeres 4 fusiform. ***Pronotum*** (Fig. 11C) about as long as wide, PL 0.47–0.55 mm, PW 0.50–0.55 mm, broadly rounded, widest at middle, finely punctate on dorsal surface, with a median and pair of lateral antebasal foveae; antebasal area strongly punctate. ***Metaventrite*** (Figs 10D, 11D) finely punctate, strongly convex, but area just above metaventral apex roundly impressed; that impression 2/3 as long as metaventral length, occupying 1/4 metaventral width; anterior margin of that impression sharply rounded, distinct. ***Elytra*** (Fig. 11E) much wider than long, EL 0.57–0.65 mm, EW 0.90–0.98 mm, trapezoidal, finely punctate, each elytron with two basal fovea; discal stria shallow, extending from basal fovea placed middle to posterior 1/3. ***Legs*.** All legs moderately short; each femora broadest near middle; protibiae, mesotibiae with dense yellow setae at apices; protibiae, metatibiae moderately straight; mesotibiae slightly arcuate at apical fourth; tarsi (Fig. 11G) each with tarsomere 2 half as long as tarsomere 3; tarsal claws (Fig. 11H) asymmetrical; anterior claws long, posterior claws thin, short. ***Abdomen*** (Fig. 11F) much wider than long, widest at tergite IV, AL 0.64–0.81 mm, AW 0.97–1.05 mm, lacking discal carinae; tergite IV longest, twice as long as V, with setose depression at base. Tergite and sternite VIII as in Fig. 13D, E. ***Aedeagus*** (Fig. 13A–C) 0.60–0.63 mm in length, well-sclerotized, slightly asymmetrical in dorsal and ventral view, tubular in lateral view; parameres symmetrical, extremely elongate, reaching apical third, each with five setae at apex; median lobe roundly curved, C-shaped in lateral view; apical part widely opened, narrowed towards basal part to connect ovoidal dorsal diaphragm; apical lobe extending downward, curved to form S-shaped in lateral view, widely opened at base, strongly produced laterally at apex; endophallus indistinct.

**Female** (Fig. 9B). BL 2.21–2.27 mm; HL 0.44–0.48 mm; HW 0.44–0.48 mm; PL 0.47–0.55 mm; PW 0.46–0.51 mm; EL 0.56–0.63 mm; EW 0.90–0.94 mm; AL 0.67–0.70 mm; AW 0.94–0.96 mm. Antennae (Fig. 10C) with antennomeres 11 simple, successively widened towards apices; antennomeres 2–10 each transverse; 11 ovoid, largest. Metaventrite convex, lacking metaventral impression.

#### Etymology.

The specific epithet refers to the strongly excavated antennae in the male of the new species.

#### Distribution.

China (Taiwan).

#### Biology.

This species was collected from leaf litter.

### Pseudophanias spinitarsis

Taxon classificationAnimaliaColeopteraStaphylinidae

Yin, Coulon & Bekchiev, 2015

9DA3D789-CFE9-5891-9A60-49816A3B1A2A

Pseudophanias spinitarsis Yin, Coulon & Bekchiev, 2015: 447.

#### Diagnosis.

This species is readily distinguished from other members of *Pseudophanias* by a combination of the following character states: Body length over 3 mm; antennal club formed by apical 4 antennomeres; antennomeres each distinctly elongate; antennomeres 8 angularly expanded laterally, 9 triangularly expanded in male; pronotal disc with conical spine; profemora concave at basal third, with bunch of thick setae; protarsomeres 2 and 3, and mesotarsomere 2 each spinose; aedeagus symmetrical, with median lobe greatly extended ventrally (Yin, Coulon and Bekchiev 2015).

### Pseudophanias yunnanicus

Taxon classificationAnimaliaColeopteraStaphylinidae

(Yin, 2019) comb. nov.

D1AC3630-14CF-5B1D-9FF7-A3D3214EC807

Chandleriella yunnanica Yin, 2019: 434.

#### Diagnosis.

This species is readily distinguished from other members of *Pseudophanias* by a combination of the following character states: Body length over 3.50 mm; antennal club formed by antennomere 11 alone; antennomere 11 strongly enlarged and modified to form bowl-like in male; aedeagus symmetric, median lobe tri-lobed at apex; parameres elongate, narrowing from base toward apex, with several long apical setae (Yin 2019).

## Discussion

Members of the supertribe Pselaphitae usually have two equal tarsal claws and have posterior claws smaller than the anterior ones in some genera. Some tribes, such as Pselaphini, have singular tarsal claws (Chandler 2001). The tribe Tyrini typically has two tarsal claws equal in size, but a few genera have protarsi with posterior tarsal claws that are reduced in size (Hlaváč and Chandler 2005). The tribe Tmesiphorini has the claw morphology similar to Tyrini (Chandler 2001), but clearly differs in the setose sulci that embrace the antennal bases. The morphology of the claws is frequently used for classification in some genera. However, interspecies differences in tarsal claw morphology have been recognized in the genus *Tmesiphorus* (Inoue et al. 2019). Although the genus *Phalepsus* Westwood of the tribe Phalepsini is distinguished from the other tribes by its strongly asymmetrical tarsal claws (Raffray 1890a; Jeannel 1949), some species have claws that are nearly subequal in size in *Phalepsus* (Chandler 2001). Therefore, tarsal claw morphology may vary at the species level within a genus in Pselaphitae.

The genus *Chandleriella* was tentatively separated from the genus *Pseudophanias* by a number of external characters (see Introduction). In this study, the three new species we placed in *Pseudophanias* show intermediate and different ratios of posterior and anterior tarsal claws. Additionally, in *Pseudophanias*, many undescribed species are recognized in Southeast Asia, and their posterior tarsal claws are reduced to various degrees in each species (Nomura pers. obs.). Therefore, the genera *Pseudophanias* and *Chandleriella* cannot be separated based on their tarsal claw morphology, and *Chandleriella*, syn. nov., is here synonymized with *Pseudophanias*. The two recognized species of *Chandleriella* are here removed to *Pseudophanias*, resulting in *P. termitophilus* (Bryant, 1915) comb. nov., and *P. yunnanicus* (Yin, 2019) comb. nov.

### List of world species

1 *Pseudophanias clavatus* Raffray, 1905: 415. Indonesia (Sumatra).

2 *Pseudophanias cribricollis* Raffray, 1895: 75. Malaysia (Penang).

3 *Pseudophanias elegans* Raffray, 1905: 413. Indonesia (Sumatra).

4 *Pseudophanias excavatus* Inoue, Nomura & Yin, sp. nov. China (Taiwan).

5 *Pseudophanias heterocerus* Raffray, 1895: 76. Singapore (Seletar).

6 *Pseudophanias malaianus* Raffray, 1890b: 214. Malaysia (Penang).

7 *Pseudophanias nakanoi* Inoue, Nomura & Yin, sp. nov. Japan (Yakushima Island; Tokara-rettô Islands).

8 *Pseudophanias pilosus* Raffray, 1895: 76. Malaysia (Penang).

9 *Pseudophanias puberulus* Raffray, 1905: 415. Malaysia (Penang).

10 *Pseudophanias punctatus* Raffray, 1905: 414. Singapore.

11 *Pseudophanias robustus* Raffray, 1905: 413. Indonesia (Sumatra).

12 *Pseudophanias spinitarsis* Yin, Coulon & Bekchiev, 2015: 447. Nepal (Pokhara).

13 *Pseudophanias termitophilus* (Bryant, 1915), comb. nov. Indonesia (Sumatra).

= *Lasinus termitophilus* Bryant, 1915: 300.

= *Chandleriella termitophila*; Hlaváč, 2000: 91.

14 *Pseudophanias tuberculatus* Raffray, 1905: 414. Indonesia (Sumatra).

15 *Pseudophanias yaimensis* Inoue, Nomura & Yin, sp. nov. Japan (Yaeyama Islands), China (Taiwan).

16 *Pseudophanias yunnanicus* (Yin, 2019), comb. nov. China (Yunnan).

= *Chandleriella yunnanica* Yin, 2019: 434.

## Supplementary Material

XML Treatment for Pseudophanias

XML Treatment for Pseudophanias yaimensis

XML Treatment for Pseudophanias nakanoi

XML Treatment for Pseudophanias excavatus

XML Treatment for Pseudophanias spinitarsis

XML Treatment for Pseudophanias yunnanicus
